# Characterization of Constituents and Anthelmintic Properties of *Hagenia abyssinica*

**DOI:** 10.3797/scipharm.1109-04

**Published:** 2012-03-02

**Authors:** Henrieke Thomsen, Katrin Reider, Katrin Franke, Ludger A. Wessjohann, Jennifer Keiser, Ermias Dagne, Norbert Arnold

**Affiliations:** 1 Leibniz Institute of Plant Biochemistry, Weinberg 3, D-06120 Halle (Saale), Germany; 2 Department of Medical Parasitology and Infection Biology, Swiss Tropical and Public Health Institute, Basel, Switzerland; 3 University of Basel, P.O. Box, CH-4003 Basel, Switzerland; 4 University of Addis Ababa, P.O. Box 30270, Addis Ababa, Ethiopia

**Keywords:** *Chaenorhabditis elegans*, Trematodes, Bioassay, Kosso, Bioactivity, Kosins

## Abstract

The dried female flowers of *Hagenia abyssinica* (Bruce) J. F. Gmel. (Rosaceae) are traditionally used as an anthelmintic remedy in Ethiopia and formerly were incorporated into the European Pharmacopoeia. One-, two- and tricyclic phloroglucinol derivatives (kosins) were suggested to be the active principles. However, polar constituents may also contribute to the activity. Therefore, we investigated for the first time the polar constituents. We isolated typical Rosaceae constituents such as quercetin 3-*O*-β-glucuronide, quercetin 3-*O*-β-glucoside and rutin. Polar kosin glycosides or derivatives could not be detected.

The anthelmintic activity of fractions of different polarity were tested against the blood fluke *Schistosoma mansoni*, the liver flukes *Clonorchis sinensis* and *Fasciola hepatica* and the intestinal fluke *Echinostoma caproni*. The anthelmintic activity decreased with increasing polarity of the tested fractions. ESI-MS investigations indicated the predominant occurrence of kosins in the active fractions.

Using the anthelmintic active extracts of *Hagenia abyssinica* we developed a simple, inexpensive bioassay against the non-parasitic nematode *Caenorhabditis elegans*, which can be used as an initial screening procedure for anthelmintic properties of crude extracts of plants or fungi. The anthelmintic activity of test extracts against the model organism was determined in a microtiter plate assay by enumeration of living and dead nematodes under a microscope.

## Introduction

*Hagenia abyssinica* (Bruce) J. F. Gmel., locally known in East and Central Africa as Kousso or Kosso, is the type species and sole congener of the monotypic genus *Hagenia* (Rosaceae). The dioecious plant is a characteristic tree of the Afro Montane Forests of Central and Eastern Africa. In Ethiopia the dried female flowers are traditionally used as an anthelmintic remedy, especially for tapeworm [1]. Infections with *Taenia saginata* Goeze are common in this region due to the regular traditional consumption of raw beef [2]. Historically it was one of the most famous African plants that were included in the European pharmacopoeia based on the description of a Portuguese priest in 1645 of the usage as vermifuge by Ethiopians [2].

**Fig. 1.**
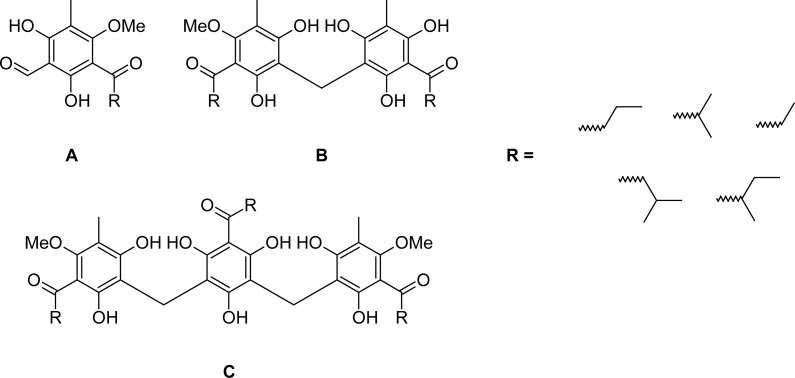
Examples for one- (A, K6), two- (B, K13) and tricyclic (C, K1) kosins

In 1870 Merck produced the first crystalline compound from Kosso, which was named “kosin” [2]. Later, it was demonstrated that kosin is an artifact generated through the alkaline extraction of the etheric crude extract of the female flowers. Several investigators proposed different structures for the active principles in Kosso [e.g. 3–6]. Finally Schiemenz and coworkers [7, 8] were able to identify the secondary metabolites in *H. abyssinica* and verify their structures by synthesis. The active pharmacological constituents are one-, two- and tricyclic phloroglucinol derivatives bearing isobutyryl, isovaleryl and 2-methylbutyryl sidechains, for historical reasons further named kosins. Examples are given in Figure 1. These compounds are similar to filix acid, the main constituent of *Dryopteris* ferns [9, 10]. Since the constituents are highly poisonous and hepatotoxic, the drug is now obsolete in Europe, even though Kosso shows remarkable anthelmintic activity and is still used widely in Ethiopia as taenicide. In addition to its anthelmintic activity kosins also show anti-cancer activity against murine adeno-carcinoma cell lines (MAC) *in vitro* and *in vivo* [11]. These cell lines behave very similarly to tumors of the human colon. Furthermore, the essential oil of *Hagenia* was shown to exhibit weak trypanocidal activity [12].

*H. abyssinica* shows a range of adverse effects. The plant possibly causes optic atrophy. A study with chicks indeed indicated retinal pathology and defects in visual behavior [13]. Tachycardia, hypotension and an enlarged liver have been reported [14]. In traditional medicine the extract is also used as abortifacient which initiated investigations examining it as a potential contraceptive agent [15].

Male flowers are reported to possess a higher emetic activity and are therefore not used as traditional medicine. However, according to chromatographic and spectroscopic studies male and female flowers are similar with respect to their content of phloroglucinols and phenolic acids [16] and also exhibit comparative toxicity to earthworms [17].

Traditionally the dried female Kosso flowers are used as decoction or suspension in water. The powdered material is usually macerated with water. Children infested with the parasite resist taking the drug and are sometimes punished by their parents in order to force them to swallow the bitter concoction. Although the nonpolar kosotoxin is believed to be the active principle, some contributing components may well exist in the crude water extract of *Hagenia abyssinica* and influence its activity [11]. The occurrence of phloroglucinol glycosides possessing a variety of biological activities have been reported from other natural sources [18]. Therefore, we investigated for the first time the polar constituents of *Hagenia abyssinica* female flowers in detail. So far only cyanidin glycosides were detected as flower pigments by TLC and HPLC [19] and the main amino acids and organic acids were described [20].

Using the anthelmintic active extracts of *Hagenia abyssinica* we developed a bioassay against the non-parasitic nematode *Caenorhabditis elegans*. In addition, the anthelmintic activity of fractions of different polarity were tested against the blood fluke *Schistosoma mansoni*, the liver flukes *Clonorchis sinensis* and *Fasciola hepatica* and the intestinal fluke *Echinostoma caproni.*

## Results and Discussion

### Characterization of constituents

The crude extract of female flowers of *Hagenia abyssinica* was successively partitioned with *n*-heptane and ethyl acetate. The remaining water layer was further separated by adsorption chromatography on Diaion HP20 resin.

**Fig. 2.**
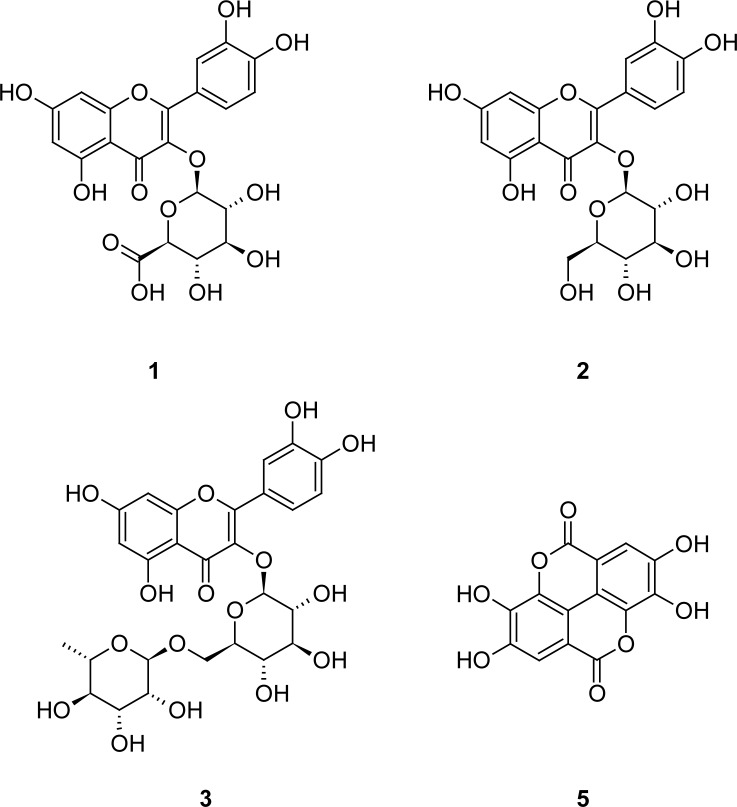
Isolated flavonoids (**1–3**) and ellagic acid (**5**) from *Hagenia abyssinica*

For identification of polar constituents the fraction eluted from Diaion with methanol was chromatographically separated resulting in the isolation of quercetin 3-*O*-β-glucuronide (**1**) [21], quercetin 3-*O*-β-glucoside (**2**) [22] and rutin (**3**) [23, 24] (Fig. 2). The isolated constituents **1**–**3** were identified by comparison of their NMR and MS data with literature. In addition, a further quercetin glycuronide (**4**) was detected, yet the position and the type of the uronic acid substituent could not be clearly determined. Compared to ^1^H and ^13^C data of **1** and according to the ^13^C NMR studies of Markham et al. [25] a substitution at position C-3’ is suggested. This is mainly indicated by shift of the signals for H-2’ and C-2’ to lower field.

In addition, ellagic acid (**5**, 300.99913 [M-H]^−^ calc. for 300.99899 C_14_H_5_O_8_) was determined in a fraction of this extract by ESI-MS investigations. However, we were not able to detect glycosides or other polar derivatives of kosins.

To our knowledge this is the first report about isolation of polar constituents of *Hagenia abyssinica*. The detected flavonoids **1–3** and ellagic acid (**5**) are typical Rosaceae constituents [26, 27].

### Development of an anthelmintic assay

Using the anthelmintic active extracts of *Hagenia abyssinica* we intended to develop a simple, inexpensive anthelmintic bioassay against the non-parasitic nematode *Caenorhabditis elegans*, which can be used as the initial screening procedure in the search for agents against parasitic helminths. Because of its short life cycle, easy maintenance and good biological characterization, *C. elegans* is a useful model organism for drug discovery and target identification [28]. Predominantly the worm is used for genetic studies. However, *C. elegans* may be also particularly useful in the development of anthelmintic drugs since it is evolutionarily closely related to parasitic worms [29]. Nevertheless, during assay development we faced several problems related to the synchronization of the worm population and the discrimination between living and dead worms. While a number of methods are described in the literature, a couple of these techniques could not be reproduced easily. For example, we could not successfully apply the colorimetric MTT-formazan assay established by James and Davey [30] for the assessment of worm viability. This assay is based on the metabolic reduction of the water-soluble yellow dye 3-(4,5-dimethylthiazol-2-yl)-2,5-diphenyltetrazolium bromide (MTT) to dark-blue water-insoluble formazan crystals, which are quantitated after extraction and dissolution in organic solvents. Because of insufficient uptake and release of the dye, long reaction times and different results depending on the commercial origin of the used MTT, we abolished this method. Furthermore, we tried to establish a viability test based on fluorescence spectroscopic measurements. Therefore the GFP-marked *C. elegans* strain TJ 356 was used. Contrary to expectations no correlation between fluorescence and vitality of the test organism could be observed [31]. Moreover, the synchronization of the worm population turned out to be a critical step. By the classical bleach procedure (hypochlorite treatment of gravid adults and hatching overnight [30, 32]) we could not achieve sufficient worm numbers to perform a screening assay.

In contrast, Lehner and coworkers [33] obtained worms of the first larval stage L1 by filtration using 10 μm mesh filters (e.g. Millipore S5EJ008M04). Since these filters are not more available nowadays we used the comparable Vectaspin3 centrifuge filter (Whatman), yet several larval stages were able to pass this filtration system. Furthermore, the nematodes were damaged during the centrifugation process and appeared raveled as a clew. Therefore, we decided to renounce the synchronization. To obtain comparable test organism nematodes were always grown 4 days on NGM agar plates. After this time period the plate is sufficiently covered with worms and only a low number of dead animals was observed.

Finally, the anthelmintic activity of extracts against the model organism *Caenorhabditis elegans* was determined in a modified microtiter plate assay by enumeration of living and dead nematodes using microscope view [34] based on an approach by Yanagida and coworkers who used a species of Diplogastridae [35, 36]. Since the automated transfer of nematodes by a worm sorter [37, 38] is too expensive and the manual picking of single animals with a worm picker under a dissection microscope is time-consuming and needs some experience, we decided to pipette a defined volume of adjusted worm suspension in the test wells. Before and after incubation with test solution, the number of living and dead animals in each well was microscopically counted. The light stimulation during the microscopic investigation induced movement of living nematodes. Therefore, agile nematodes were considered as alive, immotile animals as dead. In the next step, several DMSO concentrations were assessed to define the maximal solvent concentration tolerated by *C. elegans*. Concentrations up to 2 % DMSO did not influence the viability of the worms. However, also in control experiments up to 30 % of test worms died within a period of 30 min, probably due to drying effects caused by light during microscopic investigation. To allow an easy counting of the nematodes, only small test volumes could be used. To limit evaporation the incubation time was therefore set to 30 min. In all experiments nematodes exposed to 2 % DMSO were used as negative control. *C. elegans* incubated with the known anthelmintic ivermectin served as positive control. Incubation with 10 μg/ml ivermectin resulted in death of nearly all nematodes during the incubation time of 30 min.

#### Screening results

**Fig. 3.**
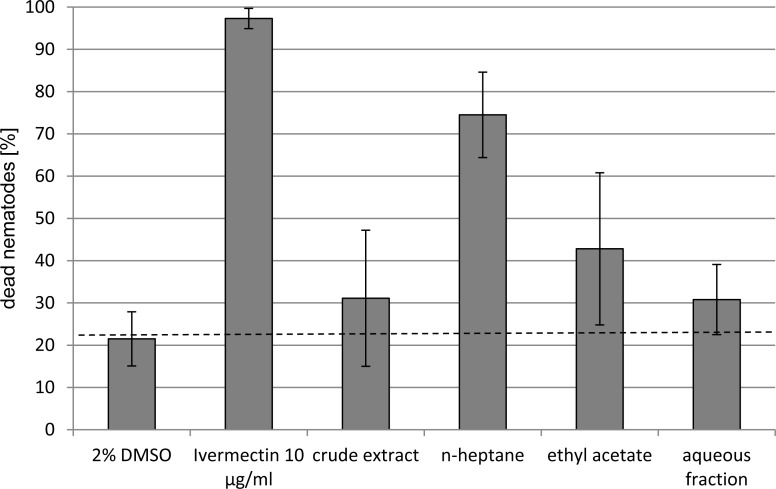
Anthelmintic activity of *Hagenia abyssinica* extracts (50 μg/ml) against *Caenorhabditis elegans*

The anthelmintic activity of the crude extract and partitioned fractions of female flowers of *Hagenia abyssinica* were tested against *Caenorhabditis elegans* (Fig. 3) and also assayed against the four trematodes *Schistosoma mansoni*, *Clonorchis sinensis, Fasciola hepatica* and *Echinostoma caproni* (Table 1).

*F. hepatica* was the least affected trematode by the test extracts. Overall, the most active trematocidal constituents seem to be present in the nonpolar fractions, whereas the polar MeOH fraction derived from separation of the water extract on Diaion HP20 is not or only slightly active. For example, while *S. mansoni* died in the presence of the crude extract, *n*-heptane and ethyl acetate extracts at 100 μg/ml within 3 h a survival of the worms of 166 h was observed in the polar MeOH fraction. One exception is the remarkable effect of the polar MeOH fraction on the intestinal fluke *E. caproni*. Note that drug susceptibility differences are common among different species and also between infective larval and adult stages. The fractions containing quercetin or ellagic acid derivatives obtained by size exclusion chromatography on Sephadex LH20 did not exhibit any anthelmintic activity (results not shown).

These results are in agreement with the phytochemical investigations. ESI-MS investigations indicated the predominant occurrence of kosins in the *n*-heptane fraction (HR-MS see Table 2), the ethyl acetate fraction contained lower amounts of kosins and in the aqueous fraction neither kosins nor polar kosin derivatives could be detected. In conclusion, the bioactivity can be attributed to the nonpolar kosins.

Contrary to our expectation, polar constituents do not contribute to the anthelmintic activity of *Hagenia abyssinica*. For the detected quercetin and ellagic acid derivatives anthelmintic properties are not described in literature. However, flavan-3-ol was previously found to have an effect on egg hatching and on the development of larvae of the sheep nematode *Trichostrongylus colubrifornis* [39].

The results of the *Caenorhabiditis* bioassay are in good correlation with the anthelmintic activities observed against the adult parasitic trematodes. Trends are evident, despite considerable variations due to biological variability and simple test conditions. Therefore, we conclude that the developed simple, fast and inexpensive test system using the model organism *C. elegans* can be applied as initial anthelmintic screening procedure. *C. elegans* can be cultivated easily and in large amounts. In contrast, adult parasitic helminthes have to be obtained by crucifying and section of host animals. Since the viability of *C. elegans* is in our test system assessed by microscopic observation, color, absorption or fluorescence of tested compounds does not influence the results. Thus, the established assay is especially useful for screening crude extracts of plants or fungi.

## Experimental

### General

The separation of extracts by column chromatography was monitored by TLC. Therefore precoated silica gel plates 60 F_254_ (Merck) were used. Spots were visualized by heating silica gel plates sprayed by vanillin-H_2_SO_4_ in MeOH. The ^1^H and ^13^C NMR spectra were recorded on a Varian Mercury 300 spectrometer at 300.22 and 75.50 MHz, respectively. Chemical shifts were referenced to internal TMS (δ = 0 ppm, ^1^H) and CDCl_3_ (δ = 77.0 ppm, ^13^C) or CD_3_OD (δ = 49.0 ppm, ^13^C), respectively. The high resolution ESI mass spectra were obtained from a Bruker Apex III Fourier transform ion cyclotron resonance (FT-ICR) mass spectrometer (Bruker Daltonics, Billerica, USA) equipped with an Infinity™ cell, a 7.0 Tesla superconducting magnet (Bruker, Karlsruhe, Germany), an RF-only hexapole ion guide and an external APOLLO electrospray ion source (Agilent, off axis spray, voltages: endplate, −3.700 V; capillary, −4.200 V; capillary exit, 100 V; skimmer 1, 15.0 V; skimmer 2, 10.0 V). Nitrogen was used as drying gas at 150 °C. The sample solutions were introduced continuously via a syringe pump with a flow rate of 120 μl/h. All data were acquired with 512 k data points and zero filled to 2048 k by averaging 32 scans. The XMASS Software (Bruker, Version 6.1.2) was used for evaluating the data.

### Plant material

Air-dried female flowers of *Hagenia abyssinica* (Bruce) J.F. Gmel. were bought at a market in Addis Ababa (Ethiopia) and determined by Prof. Ermias Dagne, Department of Chemistry, Addis Ababa University. Taxonomically the species has also been treated with the synonyms *Brayera anthelmintica* Kunth, *Banksia abyssinica* Bruce ex Steud.

### Extraction and Isolation

Dried pulverized female inflorescences (193 g) were exhaustively extracted with 80 % aqueous methanol and reduced to the aqueous phase. The crude extract was successively partitioned with *n*-heptane (11.08 g) and ethyl acetate (3.08 g). The resulting aqueous residue was further separated on a Diaion HP20 (Supelco) column eluting according to the reverse eluotropic series successively with water, methanol (6.93 g), ethyl acetate (0.05 g) and acetone containing hydrochloric acid (0.02 g).

The *n*-heptane and the ethyl acetate fraction obtained by liquid/liquid partition as well as the methanol fraction eluted from Diaion HP20 were used for anthelmintic bioassays and characterized by ESI-MS investigations.

For isolation of polar constituents 3 g of the methanol fraction were separated by column chromatography on Sephadex LH20 followed by preparative HPLC using a RP18 column resulting in the isolation of quercetin 3-*O*-β-glucuronide (**1**, 111 mg), quercetin 3-*O*-β-glucoside (**2**, 17 mg) and rutin (**3**, 6 mg) and a further quercetin glycuronide (**4**, 49 mg). In the second fraction ellagic acid (**5**) was determined by ESI-MS investigations.

#### Quercetin 3-O-β-glucuronide

*(2-(3,4-Dihydroxyphenyl)-5,7-dihydroxy-4-oxo-4H-chromen-3-yl β -*d*-glucopyranosiduronic acid,* ***1****)*

^1^H and ^13^C NMR see Bouktaib et al. [21]. HRMS: Calcd. for C_21_H_18_O_13_Na: 501.0640. Found: 501.0640.

#### Quercetin 3-O-β-glucoside

*(2-(3,4-Dihydroxyphenyl)-5,7-dihydroxy-4-oxo-4H-chromen-3-yl β -*d*-glucopyranoside,* ***2****)*

^1^H and ^13^C NMR see Zhang et al. [22]. HRMS: Calcd. for C_21_H_20_O_12_Na: 487.0847. Found: 487.0843.

#### Rutin

*(2-(3,4-Dihydroxyphenyl)-5,7-dihydroxy-4-oxo-4H-chromen-3-yl 6-O-(6-deoxy-α-*l*-mannopyranosyl)-β -d-glucopyranoside* ***3****)*

^13^C see Jerga et al. [22], ^1^H NMR see Benkiniouar et al. [24]. HRMS: Calcd. for C_27_H_30_O_12_Na: 633.1426. Found: 633.1416.

#### Quercetin glycuronide (**4**)

HRMS: Calcd. for C_21_H_17_O_13_^−^: 477.0675. Found: 477.0681. ^1^H NMR (300 MHz, CD_3_OD, TMS): δ 7.80 (brs H-2’), 7.51 (brd, J=7.0, H-6’), 6.83 (d, J=7.5, H-5’), 6.34 (s, H-8), 6.16 (s, H-6), 5.43 (brs, H-1”), 3.4-3.8 (4H). ^13^C NMR (75 MHz, DMSO-*d*6): δ 177.5 (CO), 171.9 (COOH), 164.5 (Cq), 161.2 (Cq), 156.8 (Cq), 156.5 (Cq), 148.6 (Cq), 144.9 (Cq), 133.6 (Cq), 121.3 (CH), 120.8, 117.1(CH), 115.4 (CH), 103.8, 101.8 (CH), 98.9 (CH), 93.8 (CH), 76.4 (CH), 75.2 (CH), 74.1 (CH), 71.9 (CH).

### Bioassays

#### Caenorhabditis elegans

The Bristol N2 wild type strain of *Caenorhabditis elegans* was obtained from the Caenorhabditis Genetic Center (CGC), University of Minnesota, Minneapolis, USA. The nematodes were cultured on NGM (Nematode Growth Media) petri plates at 20–23 °C using the uracil auxotroph *E. coli* strain OP50 as food source according to the methods described by Stiernagle [40]. After 4 days the nematodes were transferred to a 15 ml falcon tube by rinsing each plate twice with 2 ml M9 buffer. The worm suspension was centrifuged for 1 min at 800 G. After removal of the supernatant the nematodes were washed again with 2 ml M9 buffer under the same conditions and, depending on the amount of animals, resuspended in 2 to 8 ml M9 buffer. To this suspension 10 μl penicillin-streptomycin-solution (10 mg/ml) was added. After triply counting the nematodes in 10 μl solution droplets under a stereo microscope (Olympus SZX12) the worm number was adjusted to 20–30 animals per 20 μl. The assay was performed in 384 well plates. The outer wells were filled with water to minimize evaporation. To the test wells 20 μl worm suspension was added and the number of living and dead animals in each well were counted using the cell culture microscope Olympus CKX41. The number of living nematodes is consistent with 100 %. At staggered intervals 20 μl test solution (test compound in 4% DMSO in M9 buffer) was added followed by microscopic enumeration of living and dead test organism after 30 min incubation. Alternatively, to enhance the solubility of compounds or extracts, test solutions containing 16 % DMSO were used. Then test wells were filled with 15 μl M9 buffer, 20 μl worm suspension and 5 μl test solution. The final DMSO concentration should not succeed 2 %.

As negative and positive control always 2 % DMSO and 10 μg/ml ivermectin were used, respectively. Three replicates per test item were examined.

#### Trematodes

Extracts were tested against adult *Schistosoma mansoni*, *Echinostoma caproni*, *Fasciola hepatica* and *Clonorchis sinensis*. The studies were approved by the local veterinary agency (permit 2070) and adhered to Swiss national and cantonal regulations on animal welfare. Female NMRI mice and female Wistar rats were obtained from Harlan Laboratories (Horst, the Netherlands).

To obtain *S. mansoni*, NMRI mice were infected subcutaneously with approximately 200 *S. mansoni* cercariae. Forty-nine days post-infection mice were sacrificed with CO_2_, dissected, and all worms were recovered. Two male and two female schistosomes were placed in each well of a 48 well plate (Costar) containing 1.25 ml RPMI 1640 culture medium supplemented with 5 % fetal calf serum, 100 U/ml penicillin and 100 μg/ml streptomycin and 100 and 10 μg/ml of the test extracts. Adult *E. caproni* were collected from the excised intestines of NMRI mice 2 weeks post-infection with 50 metacercariae. Three worms were placed in each well of a 48 well plate containing 10 and 100 μg/ml of the test extracts in 1.25 ml RPMI 160 medium (supplemented with 1 % antibiotics and 1 % glucose). *Fasciola hepatica* were obtained from the local slaughterhouse. A single worm was placed in 3 ml supplemented RPMI 1640 medium and 10 and 100 μg/ml of the test extracts added. Finally, *Clonorchis sinensis* were collected from rats’ bile ducts 4 weeks post-infection with 50 metacercariae. Flukes (n=3) maintained in 1.25 ml RPMI 1640 medium (supplemented with 1% penicillin-streptomycin) were exposed to the test extracts (10 and 100 μg/ml) Control worms (untreated) were exposed to the highest concentration of DMSO (2 %) in all experiments. All worms were incubated for 180 h and the time point of death recorded. Control worms remained viable over the entire incubation period. Tests were carried out in duplicates.

## Figures and Tables

**Tab. 1.** Anthelmintic activity of *Hagenia abyssinica* extracts (100 μg/ml) against trematodes

**Organism**	**Time of death of all test organism after incubation with extracts (h)**			
	**crude extract**	***n*-heptane**	**ethyl acetate**	**MeOH (Diaion)**
*Schistosoma mansoni*	3	3	3	166
*Clonorchis sinensis*	5	5	5	48
*Fasciola hepatica*	51	17	41	>72
*Echinostoma caproni*	1	1	18	1

**Tab. 2.** ESI-FTICR-HRMS of kosins from the *n*-heptane and ethyl acetate fraction of *Hagenia abyssinica*

**Kosins**	[M-H]^−^			**structure proposal according to Schröder (1980) [41]**
	**found**	**calc. for**		
onecyclic	235.09680	C_13_H_15_O_4_^−^	235.09758	C1
	237.11250	C_13_H_17_O_4_^−^	237.11323	K8
	239.09194	C_12_H_15_O_5_^−^	239.09250	
	249.11262	C_14_H_17_O_4_^−^	249.11323	
	251.09185	C_13_H_15_O_5_^−^	251.09250	K6
	253.10742	C_13_H_17_O_5_^−^	253.10814	

twocyclic	445.18691	C_24_H_29_O_8_^−^	445.18679	K13
	447.16642	C_23_H_27_O_9_^−^	447.16606	K17, K19
	459.20173	C_25_H_31_O_8_^−^	459.20244	K2, K7, K13
	461.18181	C_24_H_29_O_9_^−^	461.18171	K19
	473.18253	C_25_H_29_O_9_^−^	473.18171	K15
	473.21705	C_26_H_33_O_8_^−^	473.21809	K2, K7, K13
	475. 19745	C_25_H_31_O_9_^−^	475.19736	K14, K18
	487.19769	C_26_H_31_O_9_^−^	487.19736	K15, K21
	487.23372	C_27_H_35_O_8_^−^	487.23374	K2,K7
	489.21290	C_26_H_33_O_9_^−^	489.21301	K14, K18
	501.21376	C_27_H_33_O_9_^−^	501.21301	K9, K15, K21
	503.22896	C_27_H_35_O_9_^−^	503.22866	K14, K18

tricyclic	639.24591	C_34_H_39_O_12_^−^	639.24470	K11
	653.26244	C_35_H_41_O_12_^−^	653.26035	K11,
	667.27778	C_36_H_43_O_12_^−^	667.27600	K1, K10, K11
	681.29365	C_37_H_45_O_12_^−^	681.29165	K1, K11, K10
	695.30817	C_38_H_47_O_12_^−^	695.30730	K4, K5, K10, K11
	709.31967	C_39_H_49_O_12_^−^	709.31708	K1, K4, K5, K10
